# Case report: Antenatal MRI diagnosis of esophageal duplication cyst

**DOI:** 10.4103/0971-3026.45350

**Published:** 2009-02

**Authors:** Rajeswaran Rangasami, Anupama Chandrasekharan, Lal Archana, Joseph Santhosh

**Affiliations:** Department of Radiology and Imaging Sciences, Sri Ramachandra Medical College and Research Institute, Sri Ramachandra University, Chennai – 600 116, India; 1 Department of Pathology, Sri Ramachandra Medical College and Research Institute, Sri Ramachandra University, Chennai – 600 116, India

**Keywords:** Antenatal USG, antenatal MRI, esophageal duplication

## Abstract

Esophageal duplication cysts are classified as a subgroup of foregut duplication cysts. They are very rare and are predominantly detected in children. Antenatal detection is very rare. We report a case of an esophageal duplication cyst that was accurately identified antenatally by USG and MRI.

Esophageal duplications are rare congenital anomalies of the alimentary tract seen in children.[1] Antenatal presentation is very rare. It is one of the causes of acute respiratory distress in infancy and childhood.[2–4] Other anomalies, especially of the nervous system, can be associated with esophageal duplication cysts. Imaging delineates the size, location, extent, and anatomic relationship of the cyst with other organs and plays a vital role in management. We report the antenatal and postnatal imaging findings in one such patient.

## Case Report

**Figure 1:**
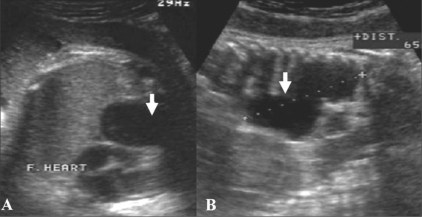
Antenatal USG: Transverse (A) and longitudinal (B) sections show a craniocaudally elongated, posterior mediastinal cyst (arrows)

A 28-year-old primigravida presented for a routine third-trimester USG examination at 38 weeks. This was her first USG study. There was no history of any significant illness in the past. The family and personal history were unremarkable. USG showed a cystic lesion in the posterior mediastinum [Figure 1]. The lesion measured 6.5 cm craniocaudally, 2.5 cm transversely, and 2.5 cm anteroposteriorly. No solid component. septations, or internal echoes were seen. The liquor was within normal limits and there was no other associated anomaly.

**Figure 2:**
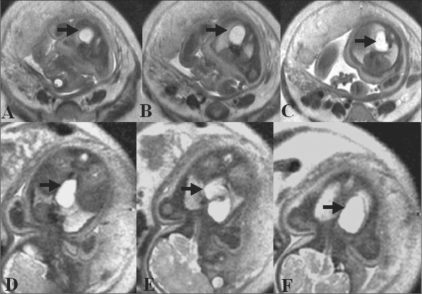
Antenatal MRI: Axial (A–C) and coronal (D–F) sections show the thoracoabdominal extent of the posterior mediastinal cyst (arrows). The lesion is in the right paravertebral region in the upper thorax and in the prevertebral region in the lower thorax

Subsequently, MRI was performed on a 1.5-T scanner using a half-Fourier acquisition single-shot turbo spin-echo (HASTE) sequence. It showed an elongated, cystic lesion in the posterior mediastinum [Figure 2]. The lesion was hyperintense on T2W images. No septations or solid components were seen. A small part of the lesion was seen to extend into the abdomen. The lesion was in the right paravertebral gutter in the upper thorax and was prevertebral in the lower thorax. There was no spinal abnormality, and no intraspinal extension was seen. A radiological diagnosis of esophageal duplication cyst was made.

**Figure 3:**
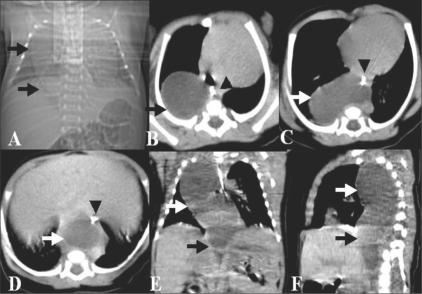
Postnatal CT scan: Scannogram (A) shows a right mediastinal opacity (arrows). Axial (B–D), coronal (E), and oblique sagittal reformatted (F) sections show the posterior mediastinal thoracoabdominal cyst (arrows). A paraesophageal course is seen. The esophagus is delineated by the feeding tube (arrowheads)

One week later the fetus was delivered by an elective Caesarean section. The neonate had persistent tachypnea. No cardiac anomaly was seen on echocardiography. A CT scan of the thorax was performed and the scannogram showed a right-sided mediastinal opacity [Figure 3A]. Axial sections showed an elongated, cystic lesion in the posterior mediastinum, extending from the thoracic inlet into the abdominal cavity up to the L_1_ level [Figure 3B–D]. The lesion was in the right paravertebral gutter in the upper thorax and was prevertebral in the lower thorax and upper abdomen. It was posterior to the esophagus in the lower thorax. No spinal anomalies were present.

**Figure 4:**
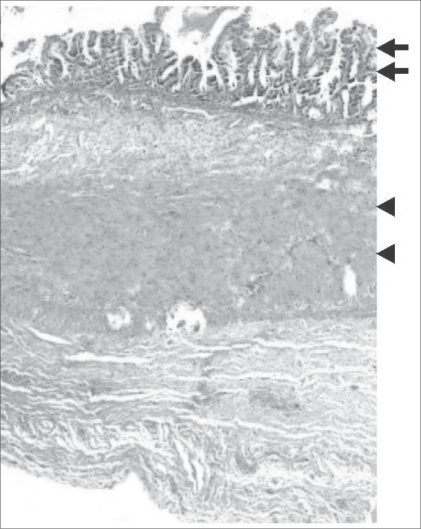
Histopathologic section of the cyst wall shows gastric-type mucosa (arrows) with a thick muscular layer (arrowheads)

On the fifth day of life, the neonate underwent a right anterolateral thoracotomy. The cyst was dissected from the adjacent tissues and totally excised. The child recovered uneventfully. Histopathological examination showed gastric-type mucosa with well-developed submucosa, muscularis propria, and serosa [Figure 4]. There was no cell atypia to suggest malignancy. These findings confirmed the radiological diagnosis.

## Discussion

Several theories have been proposed to explain the origin of duplications in the gastrointestinal tract.[1] The ‘split notochord theory’ postulated by Bentley and Smith is the most accepted explanation for foregut duplications.[1,5] During the third week of intrauterine life the notochord fuses with the embryonic ectoderm and at about the fourth week, the endoderm separates from the notochord. Duplication cysts and vertebral defects can occur if adhesions or a neuroenteric band persist after this separation.[5] When foregut cysts are associated with vertebral anomalies they are called neuroenteric cysts. Pathologically, mediastinal cysts are called esophageal duplication cysts if they are close to the esophageal wall; are covered by two muscle layers; and if the lining is squamous, columnar, cuboid, pseudostratified, or ciliated epithelium.[3,6]

An esophageal duplication cyst commonly presents with respiratory distress in infancy and childhood. It may be asymptomatic in adulthood or may present with dysphagia.[1] Bleeding can occur within the cyst when there is gastric mucosa. The cyst may enlarge in size due to retention of secretions and produce mass effect. Sometimes cysts may be complicated by malignant transformation or ulceration or they may rupture, with consequent mediastinitis and peritonitis.[7]

Of 495 duplications reviewed by Nawaz *et al*,[1] and Stringer *et al*,[8] midgut duplications accounted for 50%, foregut duplications for 36%, and hindgut duplications for 12% of cases. Esophageal duplications accounted for 19%.[8] On antenatal USG, esophageal duplication cysts are seen as posterior mediastinal cysts and appear as smooth, spherical, or tubular structures with well-defined walls. There may be layering of debris or septal folds within the cyst. The two layers of the wall–an inner echogenic mucosal layer and a hypoechoic outer muscular layer–are seen in 50% of lesions postnatally.[7] The cyst may be thoracoabdominal if very large. Antenatal MRI is very useful for further evaluation as it can show abdominal extension better. Because of the watery content of the cyst it appears hyperintense on T2W images.[2] A right-sided or posteroinferior mediastinal location favors the diagnosis of an esophageal duplication cyst.[2] The wall is relatively thicker than that of a bronchogenic cyst.[2] Continuity with the spine, if present, can also be demonstrated by MRI.

Postnatal chest radiographs may show a posterior mediastinal opacity. CT scan helps in delineating the size, location, extent, and the anatomic association of the mass to other organs. The adjacent lung can also be assessed on CT scan. MRI is also useful in characterizing the cyst and for ruling out spinal communications. The differential diagnoses include other foregut cysts, e.g., bronchogenic cyst and neuroenteric cyst.[2,9] Bronchogenic cysts are usually located around the tracheobronchial tree. Neuroenteric cysts have some communication with the spine. A well-defined, circular vertebral defect in the midst of a complex vertebral anomaly favors the diagnosis of a neuroenteric cyst[10]; this defect represents a connecting stalk between the mediastinal cyst and the intraspinal extension.

Some authors advocate only observation in smaller and asymptomatic cysts.[2] However, because of the potential for complications, complete excision of the cyst is the treatment of choice.[1]
